# Predicting cumulative incidence of adverse events in older patients with cancer undergoing first-line palliative chemotherapy: Korean Cancer Study Group (KCSG) multicentre prospective study

**DOI:** 10.1038/s41416-018-0037-6

**Published:** 2018-03-26

**Authors:** Jin Won Kim, Yun-Gyoo Lee, In Gyu Hwang, Hong Suk Song, Su Jin Koh, Yoon Ho Ko, Seong Hoon Shin, In Sook Woo, Soojung Hong, Tae-Yong Kim, Sun Young Kim, Byung-Ho Nam, Hyun Jung Kim, Hyo Jung Kim, Myung Ah Lee, Jung Hye Kwon, Yong Sang Hong, Sung Hwa Bae, Dong-Hoe Koo, Kwang-Il Kim, Jee Hyun Kim

**Affiliations:** 1 Department of Internal Medicine, Seoul National University Bundang Hospital, Seoul National University College of Medicine, Seongnam, South Korea; 2 Department of Internal Medicine, Kangbuk Samsung Medical Center, Sungkyunkwan University School of Medicine, Seoul, South Korea; 3 Department of Internal Medicine, Chung-Ang University Hospital, Chung-Ang University College of Medicine, Seoul, South Korea; 4 Department of Internal Medicine, Dongsan Medical Center, Keimyung University, Daegu, South Korea; 5 Department of Hematology and Oncology, Ulsan University Hospital, Ulsan University College of Medicine, Ulsan, South Korea; 6 Department of Internal Medicine, Uijeongbu St. Mary’s Hospital, Uijeongbu, South Korea; 7 Department of Internal Medicine, Kosin University Gospel Hospital, Busan, South Korea; 8 Department of Internal Medicine, Yeouido St. Mary’s Hospital, College of Medicine, The Catholic University of Korea, Seoul, South Korea; 9 Department of Internal Medicine, National Health Insurance Service Ilsan Hospital, Goyang, South Korea; 10 Department of Internal Medicine, Seoul National University Hospital, Seoul National University College of Medicine, Seoul, South Korea; 11 Research Institute and Hospital, National Cancer Center, Goyang, South Korea; 12 Department of Internal Medicine, Soonchunhyang University Bucheon Hospital, Soonchunhyang University College of Medicine, Bucheon, South Korea; 13 Department of Internal Medicine, Hallym University Sacred Heart Hospital, Anyang, South Korea; 14 Department of Internal Medicine, Seoul St. Mary’s Hospital, College of Medicine, The Catholic University of Korea, Seoul, South Korea; 15 Kangdong Sacred Heart Hospital, Hallym University College of Medicine, Seoul, South Korea; 16 Department of Oncology, Asan Medical Center, Ulsan University College of Medicine, Seoul, South Korea; 17 Department of Internal Medicine, Daegu Catholic University Hospital, Daegu, South Korea

**Keywords:** Chemotherapy, Outcomes research

## Abstract

**Background:**

Older patients have increased risk of toxicity from chemotherapy. Current prediction tools do not provide information on cumulative risk.

**Methods:**

Patients aged ≥ 70 years with solid cancer were prospectively enrolled. A prediction model was developed for adverse events (AEs) ≥ Grade 3 (G3), based on geriatric assessment (GA), laboratory, and clinical variables.

**Results:**

301 patients were enrolled (median age, 75 years). Median number of chemotherapy cycles was 4. During first-line chemotherapy, 53.8% of patients experienced AEs ≥ G3. Serum protein < 6.7 g/dL, initial full-dose chemotherapy, psychological stress or acute disease in the past 3 months, water consumption < 3 cups/day, unable to obey a simple command, and self-perception of poor health were significantly related with AEs ≥ G3. A predicting model with these six variables ranging 0–8 points was selected with the highest discriminatory ability (c-statistic= 0.646), which could classify patients into four risk groups. Predicted cumulative incidence of AEs ≥ G3 was discriminated according to risk groups.

**Conclusions:**

This prediction tool could identify the risk of AEs ≥ G3 after chemotherapy and provide information on the cumulative incidence of AEs in each cycle.

**Clinical Trial Id:**

WHO ICTRP number, KCT0001071

## Introduction

Older patients with cancer have distinct characteristics of physical, emotional, cognitive, and nutritional function when compared with younger patients.^1^ These patients have a decreased capacity for recovery from internal and external stress, and are susceptible to adverse events from cancer treatment.^1,2^ However, there remains minimal evidence from clinical trials on the efficacy and safety of cancer treatment in older patients.^3,4^ Therefore, it is challenging to make evidence-based decisions on the use of cytotoxic chemotherapy in older populations.

Geriatric assessment (GA) has been proved to be an objective tool to quantify the overall health status of older populations more comprehensively and precisely.^5–7^ It has been reported that GA could be associated with life expectancy, compliance of chemotherapy, postoperative mortality risk, and early death.^5,6,8,9^ Two prediction tools for chemotherapy toxicity based on GA have been reported.^10–12^ However, both of these studies were conducted in Western countries. Prevalent cancer types, drug metabolism, nutritional status, and social support are inevitably different according to different countries, races, and cultures.^13–17^ In addition, ethnic differences in drug metabolism and chemotherapy toxicities are also well established.^18,19^ Furthermore, the type and number of domains included in GA are diverse between different institutions and societies in clinical practice.^20^ Therefore, different studies using GA are needed in Asian countries. In addition, two different prediction tools for chemotherapy toxicity predicted dichotomous outcomes regardless of completed chemotherapy cycles.^10,12^ In routine clinical practice, information on the occurrence of adverse events in each chemotherapy cycle may be more useful. The cumulative incidence of toxicity may provide additional information since incidence of toxicities increase as the chemotherapy cycle proceeds.

Therefore, we aimed to develop a novel prediction tool to predict chemotherapy toxicity in Asian populations using clinical parameters and GA. The cumulative risk of toxicity was explored in proceeding chemotherapy cycles.

## Patients and methods

### Study design and participants

The Korean Cancer Study Group (KCSG) study PC13-09 was a prospective, longitudinal, and multicentre cohort study to develop a prediction tool for adverse events ≥ Grade 3 (G3) due to chemotherapy. Between February 2014 and December 2015, 301 patients were enrolled in 17 hospitals affiliated with the KCSG. The primary outcome was defined as occurrence of adverse events ≥ G3. Inclusion criteria included the following: patient ≥70 aged old; candidate for first-line palliative chemotherapy; and patients with histologically confirmed solid tumour. The exclusion criteria included the following: haematologic malignancy such as lymphoma, leukaemia, and multiple myeloma; patient who had a treatment plan to receive monotherapy with biologic agent or targeted agent, concurrent chemoradiotherapy, combination chemotherapy with investigational agents, or monotherapy with oral agents; and recurrent cases during adjuvant chemotherapy. GA was conducted after obtaining informed consent and before first-line chemotherapy. Chemotherapy regimen was chosen at an oncologist’s discretion. The dosing of chemotherapy regimen was recommended as described in the National Comprehensive Cancer Network guideline. Initial dose reduction was permitted based on clinical decision by the investigator. Adverse events were assessed by using National Cancer Institute Common Terminology Criteria for Adverse Events version 4.0 in each cycle of chemotherapy.

### Clinical parameters and GA

Pretreatment baseline measures such as laboratory findings (complete blood cell counts and chemistry), cancer type, stage, and fracture history were recorded. Chemotherapy regimen and dosing were documented. Patients were followed to collect adverse events at the end of each cycle. As in our previous studies, GA consisted of evaluating medical problems, social support, functional status, cognitive status, emotional status, nutritional status, and mobility.^5–7,21^ In brief, comorbidity was measured using the Charlson comorbidity index and was divided into low (0 points), medium (1–2 points), high (3–4 points), and very high (≥5 points) groups according to the original weighting system.^22^ Functional status was evaluated using the activities of daily living (ADL) and Korean instrument ADL (K-IADL) scores.^23–25^ At least one dependency in ADL or K-IADL was categorised as ADL-dependent or IADL-dependent, respectively. Timed Get Up and Go test (TGUG) >20 s was regarded as impaired mobility.^26^ Cognitive function was evaluated using Mini Mental Status Examination (MMSE) in the Korean version of the Consortium to Establish a Registry for Alzheimer’s disease Assessment Packet, which was divided into severe cognitive impairment (scores ≤ 16) and mild cognitive impairment (scores 17–24).^27^ For depression, Short-Form Geriatric Depression Scale scores of 5–9 and of 10 or more indicated mild depression and severe depression, respectively, (ranging from 0 to 15).^28^ In terms of nutritional status, the Mini Nutritional Assessment (MNA) score <17.0 and between 17.0 and 23.5 indicated malnutrition and a risk for malnutrition, respectively.^29^ Polypharmacy was evaluated based on number of drugs taken both descriptively and quantitatively. GA was conducted by clinical research coordinators who attended GA workshops to standardise GA and received certificate issued by KCSG.

### Development of prediction tool

Variables used to develop a predictive model for chemotherapy toxicity were as follows: clinical parameters such as age, sex, performance status, chemotherapy regimen, initial dose reduction, tumour type; laboratory findings; and all items of each domain in GA. Variables significantly associated with occurrence of adverse events ≥ G3 were identified in univariate analysis using a Cox proportional-hazards model. For developing a prediction tool, selected variables were assigned a score according to hazard ratios for adverse events ≥ G3 in a multivariate analysis of the Cox proportional-hazards model. Compared with actual incidence, the best prediction model was selected based on *c*-statistic.

### Statistical analysis

Assuming an incidence of 45% for adverse events ≥ G3 and drop-out rate of 10%, at least 200 patients were needed based on the incidence of adverse events ≥ G3 estimated in the population by Harrell’s guideline. The protocol was amended for robust significance to include 300 patients. The Cox proportional-hazards model was used to identify variables related with occurrence of adverse events ≥ G3. Multivariate models included variables that showed significance in univariate analysis with *p* < 0.05. The discriminatory ability of the prediction tool was evaluated using the *c*-statistic. Predicted probabilities for each cycle were generated based on the risk scores. This study was approved by the institutional review board of each participating centre and the KCSG (KCSG PC13-09). This study was registered with Clinical Research Information Service (WHO ICTRP number: KCT0001071). Patients completed the informed consent process.

## Results

### Patient characteristics

We enrolled 301 patients aged ≥70 years in this trial. Baseline patient characteristics, including demographics, chemotherapy, laboratory findings, and GA are shown in Tables 1 and 2. Forty-two patients (14.0%) were 80 years or older. Most patients had good performance status with Eastern Cooperative Oncology Group (ECOG) ≤ 1 (81.4%) and stage IV (97.0%). Median body mass index (BMI) was 22.5 (range 14.0–31.2). The most common cancer types were colorectal cancer (28.9%), lung cancer (24.6%), hepato-biliary-pancreatic cancer (22.3%), and stomach cancer (10.6%). In 177 patients (58.8%), initial dose reduction was applied at the first cycle.

**Table 1:** Baseline patient characteristics, clinical parameters

Variable	*N* = 301 (%)
Median age (range)	
70–79	259 (86.0)
80–89	40 (13.3)
90–100	2 (0.7)
Sex	
Male	208 (69.1)
Female	93 (30.9)
ECOG performance	
0	39 (13.0)
1	206 (68.4)
2	52 (17.3)
3/4	4 (1.3)
Cancer type	
Colorectal cancer	87 (28.9)
Lung	74 (24.6)
Hepato-biliary-pancreatic	67 (22.3)
Stomach	32 (10.6)
Urinary tract cancer (including prostate)	15 (5.0)
Head and neck	10 (3.3)
Breast	4 (1.3)
Gynaecological	4 (1.3)
Oesophageal cancer	3 (1.0)
Sarcoma	2 (0.7)
Melanoma	2 (0.7)
Thymoma	1 (0.3)
Stage	
III	7 (2.3)
IV	292 (97.0)
Unknown	2 (0.7)
Regimen	
Monochemotherapy	24 (8.0)
Combination chemotherapy	274 (91.0)
Unknown	3 (1.0)
Dose reduction (initial)	
Yes	177 (58.8)
No	119 (39.5)
Unknown	5 (1.7)
Haemoglobin, g/dL	
≥10 (female), ≥11 (male)	229 (76.1)
<10 (female), <11 (male)	72 (23.9)
Neutrophil-lymphocyte ratio^a^	
≤2.9	151 (50.2)
>2.9	150 (49.8)
Platelet, ×10^3^/µL^a^	
≥248	153 (50.8)
<248	148 (49.2)
Protein, g/dL^a^	
≥6.7	152 (50.5)
<6.7	149 (49.5)
Creatinine clearance rate^a^	
≥56.6	150 (49.8)
<56.6	151 (50.2)
Na, mmol/L	
≥135	233 (77.4)
<135	68 (22.6)
Albumin, g/dL	
≥3.6	195 (64.8)
<3.6	106 (35.2)
Cholesterol, mg/dL^a^	
≥150	142 (47.2)
<150	138 (45.8)
Unknown	21 (7.0)
C-reactive protein, mg/dL^a^	
≥1.1	150 (49.8)
<1.1	142 (47.2)
Unknown	9 (3.0)

ECOG Eastern Cooperative Oncology Group. ^a^The median value of neutrophil-lymphocyte ratio, platelet, protein, creatinine clearance rate, cholesterol, and c-reactive protein was 2.9, 248 × 10^3^/µL, 6.7 g/dL, 56.6, 150 mg/dL, and 1.1 mg/dL, respectively

**Table 2:** Baseline patient characteristics, geriatric assessment

Variable	*N* = 301 (%)
Live alone	
Yes	42 (14.0)
No	259 (86.0)
Live with spouse	
Yes	211 (70.1)
No	90 (29.9)
Previous fracture history	
Yes	12 (4.0)
No	289 (96.0)
Comorbidity (Charlson risk index)	
Low (0 points)	157 (52.2)
Medium (1–2 points)	114 (37.9)
High (3–4 points)	28 (9.3)
Very high (≥5 points)	2 (0.7)
Activity of daily living	
Independent	215 (71.4)
Dependent	86 (28.6)
Instrumental activity daily of living	
Independent	177 (58.8)
Dependent	124 (41.2)
Cognitive function (MMSE-KC)	
Intact (25–30)	134 (44.5)
Mild impairment (17–24)	137 (45.5)
Severe impairment (≤16)	30 (10.0)
Depression (SGDS)	
Intact (<5)	167 (55.5)
Mild depression (10 ≥ 5)	92 (30.6)
Severe depression) (≥10)	40 (13.3)
Unknown	2 (0.7)
Nutritional status (MNA)	
Normal (≥24)	70 (23.3)
Risk of malnutrition (17 ≤ 24)	171 (56.8)
Malnutrition (<17)	59 (19.6)
Unknown	1 (0.3)
Mobility (TGUG)	
Intact	235 (78.1)
Impaired	25 (8.3)
Unknown or not capable	41 (13.6)

*MMSE-KC* Mini Mental Status Examination in the Korean version of the Consortium to Establish a Registry for Alzheimer’s disease Assessment Packet, *SGDS* Short-Form Geriatric Depression Scale, *MNA* Mini Nutritional Assessment, *TGUG* Timed Get Up and Go test

In terms of GA, 14.0% and 70.1% of patients lived alone and with a spouse, respectively. The median number of medications taken was 5. According to Charlson comorbidity index, most patients had low or medium risks of comorbidity (52.2% and 37.9%, respectively). ADL and IADL were dependent in 28.6% and 41.2% of patients, respectively. Mild and severe impairment of cognitive function by MMSE were detected in 45.5% and 10.0% of patients, respectively. Mild and severe depression occurred in 30.6% and 13.3% of participants, respectively. The risk of malnutrition and having malnutrition, as assessed by MNA and impaired mobility by TGUG > 20 s, were identified in 56.8%, 19.6%, and 8.3%, respectively. In laboratory findings, low haemoglobin (Hb) level (Hb < 10 g/dL in female and Hb < 11 g/dL in male), hyponatremia (<135 mmol/L), and hypoalbuminemia (<3.6 g/dL) were shown in 23.9%, 22.6%, and 35.2% of patients, respectively.

### Chemotherapy and adverse events

The median number of chemotherapy cycles given was four in this study (range 25–75%, 2–7 cycles). On the discretion of the physician and according to tumour type, various chemotherapy regimens were administered in the enrolled patients (Supplementary Table 1). Five patients were not followed up for chemotherapy and adverse events. In all, 274 patients (91.0%) received combination chemotherapy and 24 patients (8.0%) received monochemotherapy. During the study period, 53.8% of patients experienced adverse events ≥ G3. Haematologic and non-haematologic adverse events ≥ G3 occurred in 37.2% and 37.9% of patients, respectively. By completion of the first chemotherapy cycle, 19.9% of patients experienced adverse events ≥ G3 (12.0% haematologic and 12.0% non-haematologic adverse events). The most common haematologic adverse events ≥ G3 were neutropaenia (28.2%), anaemia (11.6%), thrombocytopaenia (8.3%), and febrile neutropaenia (4.3%). The most common non-haematologic adverse events ≥ G3 were fatigue (7.6%), anorexia (6.3%), abdominal pain (5.0%), nausea (4.7%), and diarrhoea (3.3%) (Table 3). G5 adverse events occurred in 14 patients (4.0%), which consisted of dyspnea (3 patients), sepsis (3 patients), febrile neutropaenia (1 patient), ileus (1 patient), lung infection (1 patient), multi-organ failure (1 patient), peritoneal infection (1 patient), pneumonitis (1 patient), thromboembolic event (1 patient), and supraventricular tachycardia (1 patient). In all, 6 of 14 G5 adverse events were considered to be related with treatment according to the investigator.

**Table 3:** Common adverse events ≥ G3

Variable	*N* (%)
Haematologic adverse events, ≥G3	
Neutropaenia	85 (28.2)
Anaemia	35 (11.6)
Thrombocytopaenia	25 (8.3)
Febrile neutropaenia	13 (4.3)
Non-haematologic adverse events, ≥G3	
Fatigue	23 (7.6)
Anorexia	19 (6.3)
Abdominal pain	15 (5.0)
Nausea	14 (4.7)
Diarrhoea	10 (3.3)

### Predictive variables associated with occurrence of adverse events ≥ G3

Predictive variables were selected in the univariate analysis, which included clinical parameters such as age, sex, ECOG performance status, cancer type, chemotherapy-related variables, and each item from every domain in the GA. Six variables showed a significant association with incidence of adverse events ≥ G3. Six variables included serum protein < 6.7 g/dL, initial full-dose chemotherapy, suffering from psychological stress or acute disease in the past 3 months, water consumption of less than three cups per day, not being able to obey command of “take a piece of paper in your hand”, and self-perception of “not in good health” (Table 4).

**Table 4:** Selection of individual variables associated with occurrence of adverse events ≥ G3

Variable	Prevalence		≥G3, adverse event incidence		Univariate	
	*N* = 296^a^	%	*N* = 162	%	HR (95% CI)	*p*-value
Protein						
≥6.7	147	49.7	72	49.0	1	
<6.7	149	50.3	90	60.4	1.43 (1.05–1.95)	0.024
Initial dose reduction						
Yes	177	59.8	83	46.9	1	
No	119	40.2	79	66.4	1.66 (1.21–2.26)	0.002
Has suffered psychological stress or acute disease in the past 3 months?						
No	129	43.6	58	45.0	1	
Yes	166	56.1	104	62.7	1.49 (1.08–2.05)	0.016
How much fluid (water, juice, coffee, tea, milk…) is consumed per day?						
More than 3 cups	235	79.4	122	51.9	1	
Less than 3 cups	60	20.3	40	66.7	1.62 (1.13–2.33)	0.009
Obey command: “take a piece of paper in your hand”						
Accomplishment	242	81.8	125	51.7	1	
No accomplishment	49	16.6	34	69.4	1.49 (1.02–2.19)	0.039
Health perception						
As good or better	181	61.1	92	50.8	1	
Not as good	115	38.9	70	60.9	1.42 (1.04–1.94)	0.028

^a^Of 301 patients, 5 patients were not followed for chemotherapy and adverse events

### Developing a prediction tool for occurrence of adverse events ≥ G3

Several different models were developed based on the results in the univariate/multivariate analyses. Those six variables that showed significance in the univariate analysis were included in the multivariate analysis, with four of them remaining significant in the all multivariate models: serum protein < 6.7 g/dL; initial full-dose chemotherapy; suffering from psychological stress or acute disease in the past 3 months; and water consumption of less than three cups per day. Model 1 consists of these four variables. Models 2 and 3 were developed by adding another variable to model 1 for up to five variables. Model 4 consisted of all variables that were significant in the univariate analysis. In each model, scores for each variable were assigned based on hazard ratio in the multivariate analysis (Table 5).

**Table 5:** Modeling of prediction tool

Variable	Model 1			Model 2			Model 3			Model 4		
	Multivariate			Multivariate			Multivariate			Multivariate		
	HR (95% CI)	*p*-value	Score	HR (95% CI)	*p*-value	Score	HR (95% CI)	*p*-value	Score	HR (95% CI)	*p*-value	Score
Protein												
≥6.7	1			1			1			1		
<6.7	1.44 (1.05–1.97)	0.024	1	1.38 (1.01–1.90)	0.047	1	1.42 (1.03–1.94)	0.032	1	1.35 (0.98–1.86)	0.067	1
Initial dose reduction												
Yes	1			1			1			1		
No	1.74 (1.27–2.38)	<0.001	2	1.75 (1.28–2.40)	<0.001	2	1.72 (1.26–2.35)	<0.001	2	1.72 (1.25–2.37)	<0.001	2
Has suffered psychological stress or acute disease in the past 3 months?												
No	1			1			1			1		
Yes	1.47 (1.06–2.03)	0.016	1	1.47 (1.06–2.05)	0.022	1	1.38 (0.98–1.94)	0.063	1	1.38 (0.97–1.96)	0.071	1
How much fluid (water, juice, coffee, tea, milk…) is consumed per day?												
More than 3 cups	1			1			1			1		
Less than 3 cups	1.57 (1.09–2.26)	0.015	2	1.55 (1.06–2.25)	0.023	2	1.58 (1.10–2.28)	0.015	2	1.56 (1.07–2.27)	0.021	2
Obey command: “take a piece of paper in your hand”												
Accomplishment				1						1		
No accomplishment				1.32 (0.90–1.95)	0.162	1				1.33 (0.90–1.97)	0.147	1
Health perception												
As good or better							1			1		
Not as good							1.27 (0.87–1.70)	0.246	1	1.22 (0.87–1.71)	0.243	1

To select the best model for the prediction of adverse events ≥ G3 with the highest discriminatory ability, *c*-statistics were calculated. Model 4 showed the highest mean *c*-statistic (0.646, Supplementary Table 2). Finally, model 4, consisting of six variables, was confirmed as the most accurate prediction tool for occurrence of adverse events ≥ G3 (Supplementary table 3).

### Prediction tool for occurrence of adverse events ≥ G3

**Fig. 1:**
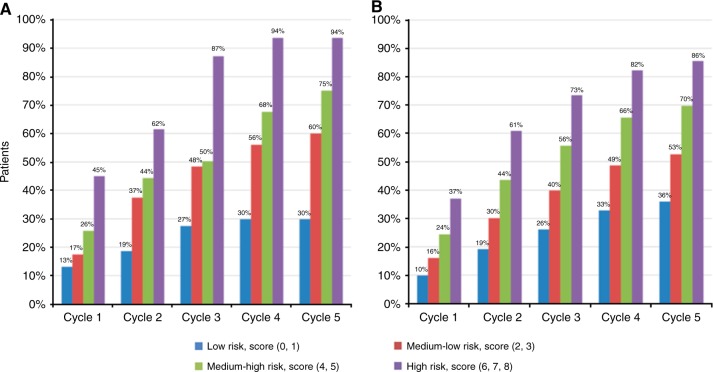
Actual incidence (**a**) and predicted incidence (**b**) of adverse events ≥ G3 according to the risk group and cycle

The predication tool indicated scores ranging 0–8 point. Patients with higher scores had more risk for adverse events ≥ G3. Score distribution is indicated in supplementary figure 1. Risk groups were classified according to score as low risk (0, 1), medium-low risk (2, 3), medium-high risk (4, 5), and high risk (6, 7, 8). Of patients classified by risk groups, 61 (21.0%), 143 (49.3%), 66 (22.8%), and 20 (6.9%) were in low, medium-low, medium-high, and high-risk groups, respectively. In each risk group, predicted cumulative incidence of adverse events ≥ G3 increased with the chemotherapy cycle number. In the low-risk group, predicted cumulative incidence of adverse events ≥ G3 was 9.9%, 19.2%, 26.1%, 32.8%, and 35.9% in cycle 1, cycle 2, cycle 3, cycle 4, and cycle 5, respectively. In the medium-low-risk group, predicted cumulative incidence of adverse events ≥ G3 was 16.0%, 30.1%, 39.8%, 48.7%, and 52.6% in cycle 1, cycle 2, cycle 3, cycle 4, and cycle 5, respectively. In the medium-high-risk group, predicted cumulative incidence of adverse events ≥ G3 was 24.4%, 43.6%, 55.6%, 65.5%, and 69.7% in cycle 1, cycle 2, cycle 3, cycle 4, and cycle 5, respectively. In the high-risk group, predicted cumulative incidence of adverse events ≥ G3 was 37.0%, 60.9%, 73.4%, 82.3%, and 85.5% in cycle 1, cycle 2, cycle 3, cycle 4, and cycle 5, respectively. In the same cycle, predicted cumulative incidence of adverse events ≥ G3 was also discriminated according to risk group (Fig. 1).

## Discussion

From this prospective longitudinal multicentre study, we developed a novel prediction tool to identify those patients at risk for adverse events ≥ G3 in older patients undergoing first-line chemotherapy. This prediction tool consisted of six variables from clinical parameters and GA conducted pre-chemotherapy, with an ability to identify four risk groups (low, medium-low, medium-high, and high), which quantified cumulative risk rates of adverse events ≥ G3 from the first to the fifth cycle.

There are two established and validated prediction tools for severe chemotherapy toxicity (G4 haematologic toxicity and G3/4 non-haematologic toxicity; G3-5 chemotherapy-related toxicity).^10–12^ Nevertheless, our prediction tool has distinct features in terms of study population, primary outcome, and modeling methods as compared to previous studies.

This study population consisted of Koreans and patients initiating first-line chemotherapy. Previous studies were conducted in hospitals of the United States with most patients of Caucasian race. Toxicity profiles from chemotherapy are reported to be different by geographic regions due to ethnic differences in drug metabolism, nutritional status, social support, and medical culture.^19,30^ For example, Asians showed more frequent occurrence of febrile neutropaenia (18.6% vs 7.1%), edema (26.1% vs 5.4%), myalgia (42.3% vs 14.7%), and decreased appetite (47.0% vs 19.1%) in the phase III CLEOPATRA trial.^30^

Moreover, our study population showed a lower median BMI (22.5 vs. 25.9 in a previous study conducted in the United States),^10^ included more gastrointestinal cancer types, more frequent dose reductions (58.8% vs 24.0%) as compared with previous study. The different population characteristics and tolerances to chemotherapy justify the development chemotherapy prediction tools developed in Asian populations. Furthermore, patients in this study who were initiating only first-line chemotherapy were enrolled in contrast with previous studies, which allowed prior palliative chemotherapy. Vulnerability for chemotherapy could be different across chemotherapy lines. With additional lines of chemotherapy, chemotherapy toxicity may occur more frequently. It would be more ideal to include homogenous populations in terms of number of chemotherapy lines to develop precise chemotherapy toxicity prediction tool.

Regarding the primary outcome, in contrast with other studies using treatment-related toxicity, our study used adverse event as the primary measure regarding outcome. It is often difficult to determine the causality of adverse events in clinical practice. In view of older patients with cancer and their family members, events itself during chemotherapy are important regardless of causality. Therefore, adverse event is a suitable outcome measure in this study with older patients with cancer.

In terms of modeling methods, our prediction models were developed using the Cox proportional-hazards model, in which applied chemotherapy cycles and cycle with first adverse event ≥ G3 were incorporated. Therefore, cumulative incidence of adverse event ≥ G3 could be suggested on the contrary to generating just dichotomous outcomes in previous studies. This point is important because incidence of adverse events increase inevitably with proceeding of chemotherapy cycles and most patients recover from adverse event ≥ G3 and continue the next cycle. In our study, 45% of patients in the high-risk group were expected to experience adverse events ≥ G3 during the first cycle. However, almost all patients in the high-risk group were expected to experience adverse events ≥ G3 within fourth cycle. Meanwhile, no more than 30% of patients in the low-risk group were expected to have adverse events ≥ G3 as the cycles proceeded. This information might be valuable to decide and discuss chemotherapy application with older patient with cancer and their family members.

Finally, our prediction tool can utilise a questionnaire of only six questions, which would allow for a simple clinical application in busy oncology clinics. Six questions were related to nutritional status (two questions), recent illness (one question), chemotherapy dosing (one question), cognitive function (one question), and self-estimation for health status (one question). These components were reported as important factors in previous studies associated with geriatric outcome.^6,10,12,21^ In previous studies for the prediction of chemotherapy toxicity, chemotherapy dosing, nutritional status, and cognitive function were also included.^10,12^ Our prediction tool suggested cumulative incidence of adverse events ≥ G3 in each cycle of first-line chemotherapy with a mean *c*-statistic of 0.646 to predict adverse events ≥ G3, which is comparable to discriminative power found in previous studies.^10–12^

There are some limitations to this study. First, the applied chemotherapy regimens and cancer types were heterogeneous. It would be ideal to conduct this study in a specific tumour type, being treated with a specific chemotherapy regimen. However, our study aimed to determine common geriatric factors that affect the occurrence of adverse events ≥ G3. Compared with previous studies, only patients who would receive first-line chemotherapy were enrolled for a more homogenous study population. Second, G2 adverse events are also important in vulnerable older patients with cancer who are receiving chemotherapy. Hospitalisation, laboratory abnormality or symptoms etc. to stop chemotherapy, and mortality during chemotherapy could be a good outcome measure. However, these measures are mostly covered in adverse events ≥ G3, which were defined as the primary outcome in this study. Third, this study was performed in Korean patients and patients with first-line chemotherapy, but external validation in different populations or other Asian countries should be conducted. Fourth, in this study population, the risk scores of previous tools could not be calculated due to discrepancy in domains used in GA across studies. Direct comparison of efficacy of this tool with previous tools could not be performed. This prediction tool was designed in different population from previous studies, such as clinically homogeneous and Asian population. Furthermore, cumulative incidence showed in our prediction tool could give another information to clinic practice. Therefore, it would be worth to develop this tool regardless of comparison of efficacy with previous tools.

We developed simple, six-item novel prediction tool for adverse events ≥ G3, which would be easier to use in daily practice and which could provide patients and physicians information to plan chemotherapy in an Asian population. In high-risk patients, a high incidence of adverse events should be anticipated, and preventive and proactive measures should be administered. In other hands, active chemotherapy could be encouraged for patients in the low-risk group. Future studies are needed to evaluate geriatric intervention in high-risk patients to promote the safe use of chemotherapy in older patients with cancer.

## Electronic supplementary material

supplementary tables figure
