# Supplementary material for: Predicting cumulative incidence of adverse events in older patients with cancer undergoing first-line palliative chemotherapy: Korean Cancer Study Group (KCSG) multicentre prospective study
Source: Br J Cancer. 2018 Mar 26;118(9):1169–75. doi: 10.1038/s41416-018-0037-6 (PMC5943243; doi:10.1038/s41416-018-0037-6)

Supplementary table 1. Chemotherapy regimen

| Cancer type | N |
| --- | --- |
| Head and neck |  |
| 5-Fluorouracil/Cisplatin | 5 |
| Docetaxel/Cisplatin | 3 |
| Cetuximab/5-Fluorouracil/Cisplatin | 1 |
| Unknown | 1 |
| Esophageal cancer |  |
| 5-Fluorouracil/Cisplatin | 3 |
| Stomach cancer |  |
| 5-Fluorouracil/Oxaliplatin/Leucovorin | 16 |
| Capecitabine/Oxaliplatin | 7 |
| Trastuzumab/ Capecitabine/Cisplatin | 3 |
| Capecitabine/ Cisplatin | 2 |
| 5-Fluorouracil/Etoposide/Cisplatin | 2 |
| Etoposide/Cisplatin | 1 |
| Bevacizumab/5-Fluorouracil/Irinotecan/Leucovorin | 1 |
| Colorectal cancer |  |
| 5-Fluorouracil/Irinotecan/Leucovorin | 32 |
| Bevacizumab/5-Fluorouracil/Irinotecan/Leucovorin | 26 |
| Cetuximab/5-Fluorouracil/Irinotecan/Leucovorin | 14 |
| 5-Fluorouracil/Oxaliplatin/Leucovorin | 9 |
| Bevacizumab/5-Fluorouracil/Oxaliplatin/Leucovorin | 1 |
| 5-Fluorouracil/Leucovorin | 1 |
| Capecitabine/Oxaliplatin | 1 |
| Paclitaxel/Carboplatin | 1 |
| Etoposide/Cisplatin | 1 |
| Unknown | 1 |
| Hepato-biliary-pancreatic |  |
| Gemcitabine/Cisplatin | 38 |
| 5-Fluorouracil/Irinotecan/Oxaliplatin/Leucovorin | 8 |
| Gemcitabine | 6 |
| Gemcitabine/Erlotinib | 5 |
| Gemcitabine/Oxaliplatin | 2 |
| Etoposide/Cisplatin | 1 |
| Etoposide/Carboplatin | 1 |
| Gemcitabine/Oxaliplatin/Erlotinib | 1 |
| 5-Fluorouracil/Leucovorin | 1 |
| 5-Fluorouracil/Oxaliplatin/Leucovorin | 1 |
| Pemetrexed/Cisplatin | 1 |
| Capecitabine/Oxaliplatin | 1 |
| Unknown | 1 |
| Gynecological |  |
| Paclitaxel/Carboplatin | 3 |
| Docetaxel/Cisplatin | 1 |
| Sarcoma |  |
| Paclitaxel | 2 |
| Urinary tract cancer (including -prostate) |  |
| Gemcitabine/carboplatin | 8 |
| Gemcitabine/cisplatin | 5 |
| Docetaxel | 2 |
| Lung |  |
| Pemetrexed/Cisplatin | 19 |
| Etoposide/Cisplatin | 16 |
| Gemcitabine/Cisplatin | 7 |
| Gemcitabine/Carboplatin | 7 |
| Etoposide/ Carboplatin | 5 |
| Docetaxel/Cisplatin | 5 |
| Gemcitabine | 4 |
| Docetaxel/ Carboplatin | 3 |
| Etoposide/Carboplatin/Ifosfamide | 3 |
| Docetaxel | 2 |
| Pemetrexed/Carboplatin | 1 |
| Paclitaxel/Cisplatin | 1 |
| Bleomycin/Etoposide/Cisplatin | 1 |
| Breast |  |
| Doxorubicin/Cyclophosphamide | 2 |
| Docetaxel/Cisplatin | 1 |
| Docetaxe/Doxorubicin/Cyclophosphamide | 1 |
| Melanoma |  |
| Dacarbazine | 1 |
| Dacarbazine/Cisplatin | 1 |
| Thymoma |  |
| Doxorubicin/Cisplatin/Vincristine/ Cyclophosphamide | 1 |

Supplementary table 2. Model selection of prediction model

| C statistics (95% CI) | Cycle 1 | Cycle 2 | Cycle 3 | Cycle 4 | Cycle 5 | Mean value |
| --- | --- | --- | --- | --- | --- | --- |
| Model 1 | 0.636 (0.556 - 0.717) | 0.621 (0.559 - 0.684) | 0.618 (0.561 - 0.675) | 0.627 (0.573 - 0.682) | 0.629 (0.575 - 0.683) | 0.626 |
| Model 2 | 0.648 (0.567 - 0.729) | 0.635 (0.572 - 0.699) | 0.629 (0.572 - 0.687) | 0.637 (0.583 - 0.692) | 0.639 (0.586 - 0.693) | 0.638 |
| Model 3 | 0.646 (0.567 - 0.725) | 0.634 (0.572 - 0.696) | 0.629 (0.572 - 0.685) | 0.636 (0.582 - 0.689) | 0.638 (0.585 - 0.691) | 0.637 |
| Model 4 | 0.657 (0.577 - 0.737) | 0.645 (0.582 - 0.707) | 0.637 (0.580 - 0.694) | 0.644 (0.590 - 0.698) | 0.646 (0.592 - 0.699) | 0.646 |

Supplementary table 3. Prediction tool for occurrence of adverse events ≥ G3

| **Variables** | | **Score** |
| --- | --- | --- |
| **Protein level** | ≥ 6.7 | 0 |
|  | < 6.7 | 1 |
| **Initial dose reduction** | Yes | 0 |
|  | No | 2 |
| **Has suffered psychological stress or acute disease in the**  **past 3 months?** | No | 0 |
|  | Yes | 1 |
| **How much fluid (water, juice, coffee, tea, milk...) is consumed per day?** | More than 3 cups | 0 |
|  | Less than 3 cups | 2 |
| **Obey command :**  **“Take a piece of paper in your hand ”** | Accomplishment | 0 |
|  | No accomplishment | 1 |
| **How about your health status? (Health perception)** | As good or better | 0 |
|  | Not as good | 1 |
| **Total score** | | 8 |

Supplementary figure 1. Distribution of scores with the prediction tool


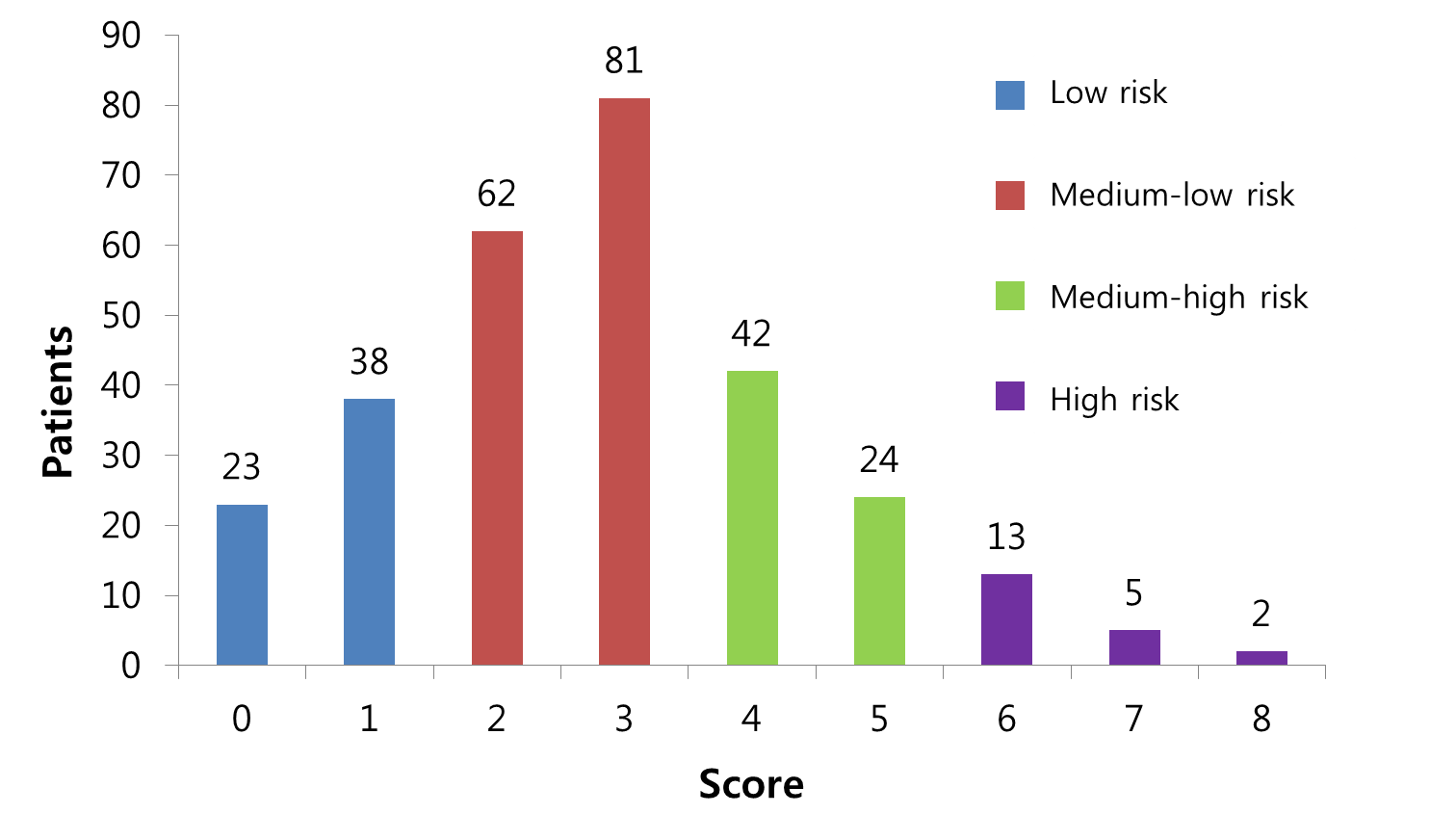

Supplement: Supplementary file 1 — supplementary tables figure [file 41416_2018_37_MOESM1_ESM.docx]
